# The changing scenario of nephron sparing approaches to treat renal tumors: Making a case to save the nephrons!

**DOI:** 10.4103/0970-1591.57920

**Published:** 2009

**Authors:** Krishnanath Gaitonde

**Affiliations:** Assistant Professor of Urology, Codirector of Endourology, Laparoscopy and Robotic Surgery Fellowship Program, University of Cincinnati College of Medicine, Cincinnati, Ohio, USA. E-mail: gaitonk@uc.edu

There is an increasing incidence of renal cell cancer being noted worldwide. In the United States, an estimated 57,760 new cases of cancer of the kidney and renal pelvis will be diagnosed in 2009, an increase of 3000 cases compared to the previous year.[1,2] An overwhelming majority of these tumors will be renal cell carcinomas. The widespread use and application of diagnostic imaging modalities such as computerized tomography (CT) has resulted in an increased number of incidentally detected small renal masses. T1a tumors (less than 4 cm) account for the largest proportion of newly diagnosed renal cancers today.[3]

Most of these renal tumors are amenable to nephron sparing approaches, resulting in maximal preservation of renal parenchyma with comparable oncologic outcomes to radical nephrectomy,[4] which was traditionally considered the standard management for all renal tumors. Several recent studies have also highlighted the overall survival benefit of partial nephrectomy over radical nephrectomy.[5–7] This has been attributed to the development of chronic renal insufficiency, and related cardiovascular events and death in patients who underwent radical nephrectomy. Despite the overwhelming evidence in favor of partial nephrectomy, many patients still receive radical nephrectomy for small renal tumors, which may be amenable to nephron sparing surgery. Population based studies in the United States have shown that partial nephrectomy is offered to only about 20% of patients with T1a renal tumors less than 4 cm.[8] This trend does seem to be changing, especially in tertiary referral centers.[9]

With the changing trend of decreasing size of these incidentally detected renal masses, there has been an explosion of newer minimally invasive treatment approaches, with the common goal being renal function preservation while maintaining established oncologic outcomes. Open partial nephrectomy, which has stood the test of time, is the benchmark against which the outcomes of all these newer techniques have to be measured. Laparoscopic partial nephrectomy and robotic partial nephrectomy are both surgical advances in the technique of performing partial nephrectomy, with the inherent significant advantages of minimally invasive surgery such as decreased pain and improved cosmesis. Renal tumor ablation therapy, on the other hand, represents a paradigm shift in the surgical thought process, since it involves in vivo destruction of tumor tissue without actually excising it from within the kidney. Tumor ablation can be accomplished clinically by freezing (cryoablation) or using heat energy (radiofrequency ablation) to destroy the tumor tissue. Other energy modalities being evaluated include high intensity focused ultrasound (HIFU).

Renal ablative procedures can be performed laparoscopically or percutaneously (in selected cases) using real time CT or ultrasound image guidance. Renal ablation therapy is an exciting new therapeutic approach which is poised to establish its role in the urologist's armamentarium for dealing with renal cancer as scientific data gradually emerges about the long term outcomes of its efficacy. Till such time, it should be used in a judicious manner in carefully selected patients, after obtaining informed consent about the limited long term data about oncologic outcomes.

The next important question which needs to be answered is whether all incidentally detected renal tumors should be treated with a ‘one size fits all’ approach, given that a proportion of these lesions will be benign and most will exhibit a slow rate of growth. This has resulted in the evolution of watchful waiting or active surveillance as a viable alternative in certain clinical situations such as the elderly patient or high-risk surgical candidate. The risk of metastases during watchful waiting for small renal masses has been estimated to be about 1%.[10] Percutaneous tumor biopsy, which seems to be experiencing a gradual clinical comeback, could play a significant role in this setting with its potential for pretreatment histopathologic characterization and molecular profiling to predict the oncologic aggressiveness of these lesions. Molecular profiling of renal tumors represents a new frontier in oncology and could help the urologist to tailor the management approach to fit the expected clinical behavior of the tumor - so called ‘personalized medicine’. Further research in this field is warranted before it can be applied in a clinical setting.

The contributing authors to this symposium have done an excellent job in providing information about the various nephron sparing approaches for managing renal tumors. They are all leaders in their respective areas of urologic expertise and I am extremely grateful to them for their time and commitment in providing a well balanced overview of the smorgasbord of treatment options available to the urologist when dealing with renal tumors. Before I conclude, I would like to re-emphasize the importance of sparing nephrons, irrespective of whether this is achieved with an open or minimally invasive technique. When faced with a tumor which could be difficult to treat with laparoscopic or robotic partial nephrectomy but possible to remove using open partial nephrectomy, it is important to avoid the temptation of alternatively offering a laparoscopic radical nephrectomy to the patient only for the benefits of the minimally invasive approach (decreased pain, better cosmesis).[11] Responsible application of newer technologies is what will allow us to achieve optimal oncologic and functional outcomes in our patients. As the English poet Alexander Pope said “*be not the first by whom the new is tried, nor yet to lay the old aside*”.
